# Poly[μ-aqua-diaqua­(μ_3_-*N*′-carboxy­methyl­ethylenediamine-*N*,*N*,*N*′-tri­acetato)oxidopotassium(I)vanadium(IV)]

**DOI:** 10.1107/S1600536808017030

**Published:** 2008-06-07

**Authors:** Rui-Hong Zhang, Li-Ping Lu, Ming-Xia Li, Miao-Li Zhu

**Affiliations:** a Institute of Molecular Science, Key Laboratory of Chemical Biology and Molecular Engineering of the Education Ministry, Shanxi University, Taiyuan, Shanxi 030006, People’s Republic of China

## Abstract

In the crystal structure of the title compound, [KV(C_10_H_13_N_2_O_8_)O(H_2_O)_3_]_*n*_, the V^IV^ ion adopts a distorted octa­hedral geometry, coordinated by one oxide group, two N and three carboxylate O atoms from the same *N*′-carboxy­methyl­ethyl­ene­diamine-*N*,*N*,*N*′-triacetate (HEDTA) ligand. The potassium ion is hepta­coordinated by two water mol­ecules, two bridging water mol­ecules and three carboxylate O atoms from three neighbouring HEDTA ligands. The HEDTA ligands and some of the water mol­ecules act as bridges, linking the compound into a three-dimensional architecture *via* 2_1_ screw, *c*-glide, translation and inversion symmetry operators. Meanwhile, three types of O—H⋯O hydrogen bonds provide an additional stabilization of the three-dimensional architecture.

## Related literature

For related literature, see: Crans *et al.* (2004 ▶); Khanra *et al.* (2007 ▶); Tsuchida *et al.* (1999 ▶).
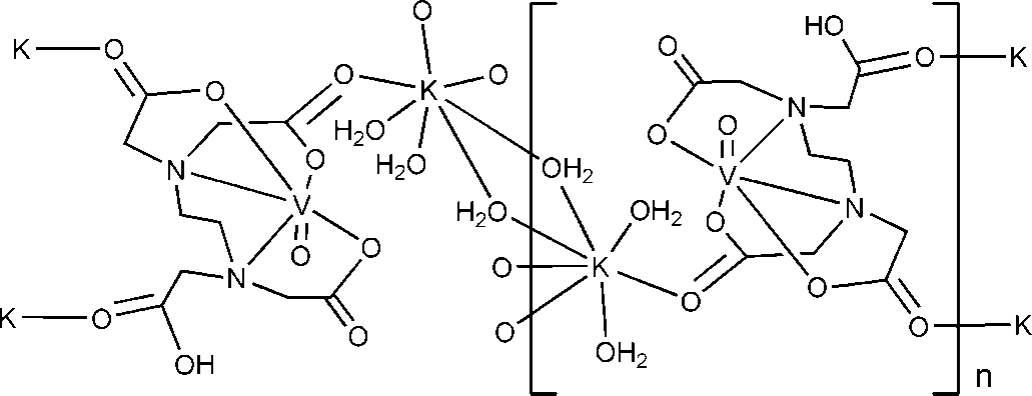


## Experimental

### 

#### Crystal data

[KV(C_10_H_13_N_2_O_8_)O(H_2_O)_3_]
*M* *_r_* = 449.31
Monoclinic, 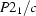

*a* = 6.6701 (13) Å
*b* = 13.618 (3) Å
*c* = 18.693 (4) Å
β = 96.150 (2)°
*V* = 1688.2 (6) Å^3^
*Z* = 4
Mo *K*α radiation
μ = 0.90 mm^−1^
*T* = 298 (2) K
0.40 × 0.30 × 0.20 mm

#### Data collection

Bruker SMART 1K CCD diffractometer
Absorption correction: multi-scan (*SADABS*; Sheldrick, 2000 ▶) *T*_min_ = 0.714, *T*_max_ = 0.840
6813 measured reflections
2957 independent reflections
2613 reflections with *I* > 2σ(*I*)
*R*_int_ = 0.024

#### Refinement

*R*[*F* ^2^ > 2σ(*F* ^2^)] = 0.036
*wR*(*F* ^2^) = 0.088
*S* = 1.07
2957 reflections
236 parameters
H-atom parameters constrained
Δρ_max_ = 0.33 e Å^−3^
Δρ_min_ = −0.22 e Å^−3^

### 

Data collection: *SMART* (Bruker, 2000 ▶); cell refinement: *SAINT* (Bruker, 2000 ▶); data reduction: *SAINT*; program(s) used to solve structure: *SHELXS97* (Sheldrick, 2008 ▶); program(s) used to refine structure: *SHELXL97* (Sheldrick, 2008 ▶); molecular graphics: *SHELXTL/PC* (Sheldrick, 2008 ▶); software used to prepare material for publication: *PLATON* (Spek, 2003 ▶) and *publCIF* (Westrip, 2008 ▶).

## Supplementary Material

Crystal structure: contains datablocks I, global. DOI: 10.1107/S1600536808017030/fj2120sup1.cif

Structure factors: contains datablocks I. DOI: 10.1107/S1600536808017030/fj2120Isup2.hkl

Additional supplementary materials:  crystallographic information; 3D view; checkCIF report

## Figures and Tables

**Table 1:** Hydrogen-bond geometry (Å, °)

*D*—H⋯*A*	*D*—H	H⋯*A*	*D*⋯*A*	*D*—H⋯*A*
O8—H8⋯O3^i^	0.82	1.75	2.542 (3)	162
O12—H12*B*⋯O11^ii^	0.82	2.03	2.802 (3)	157
O11—H11*B*⋯O4^iii^	0.82	2.17	2.960 (3)	162
O10—H10*B*⋯O6^iii^	0.82	2.20	2.987 (3)	161
O12—H12*A*⋯O5^iv^	0.82	1.99	2.804 (3)	169
O11—H11*A*⋯O7^ii^	0.82	1.99	2.801 (3)	169
O10—H10*A*⋯O12^v^	0.82	2.26	2.983 (3)	147

Symmetry codes: (i) 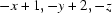; (ii) 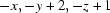; (iii) 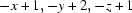; (iv) 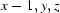; (v) 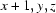.

**Fig. 1.**
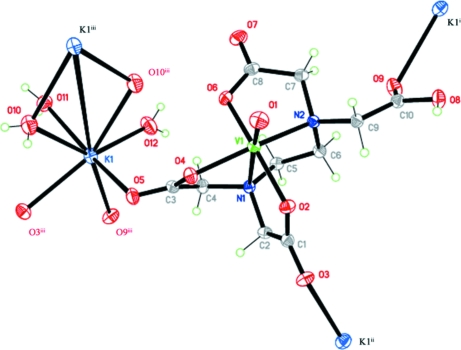
A view of the structure of (I) with displacement ellipsoids drawn at the 30% probability level. Symmetry codes: i -x, y + 1/2, -z + 1/2; ii x, -y + 3/2, z - 1/2; iii -x, -y + 2, -z

**Fig. 2.**
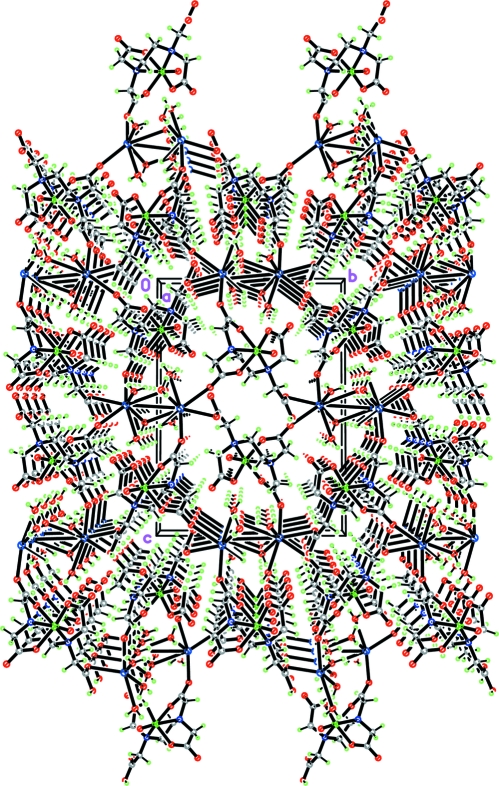
The packing view in the title complex (I).

## supplementary crystallographic information

### Comment

The vanadium complexes have been attracted great attention because of their versatile properties including biological activities(Crans *et al.*, 2004), magnetic property(Khanra *et al.*, 2007), catalytic abilities (Tsuchida *et al.*, 1999) and so on. Especially, we are interested in the protein tyrosine phosphatase 1B (PTP1B) inhibition activity of vanadium compounds. Thus, the title compound (I) was synthesized and its crystal structure is reported here.

The X-ray crystallographic analysis shows that there are two metal ion centres in the asymmetric unit of the title compound(Fig 1). V^IV^ adopts a six coordinated geometry consisting of a O atom(O1) from vanadyl, two N and three carboxyl O atoms(O2, O4 and O6) from same symmetric edta ligand while potassium is hepta-coordinated by two water molecules, two bridging water molecules and three carboxyl O atoms (O3, O5 and O9) respectively from three neighbouring edta ligands with different symmetry. Each edta ligand acts as a bridge simultaneously coordinating to three neighbouring K^+^ ions while coordinating to one vanadium. Neighbouring K^+^ ions are bridged through two coordinated water molecules(O10). As the result of these coordination, the compound is constructed to three-dimensional structure by O9 atom *via* 2_1_-screw, O3 *via* c-glide & translation and K1 *via* inversion & translation(Fig 2). Meanwhile, three types of O—H···O hydrogen bonds (Table 1) take part in the stabilization of the three-dimensional architecture(Fig 2). The first type is the coordination water O atoms (O10, O11 and O12) acting as H donors while carboxyl O atoms(O4, O5, O6 and O7) of edta ligands as acceptors. The second is between coordination water molecules[O12—H12B···O11(-*x*, 2 - *y*, 1 - *z*) and O10—H10A···O12(1 + *x*, *y*, *z*)]. The third type of O8—H8···O3(1 - *x*, 2 - *y*, -*y*) hydrogen bond joins neighbouring edta ligands.

### Experimental

All chemicals were of reagent grade, were commercially available and were used without further purification. H4EDTA(11.69 g, 40 mmol) was added to 100 ml of water and neutralized with 11.20 g (80 mmol) of Potassium carbonate. 6.52 g (40 mmol) of VOSO_4_ was added to the solution, stirred for 24 h. Evaporation of the solution using a rotary evaporator was concentrated to 20 ml, then the solution with blue flocculent crystals was filtered, The blue crystals were obtained by slow evaporation of the solvent about two days at room temperature.

### Refinement

H atoms attached to C and O(EDTA) atoms of (I) were placed in geometrically idealized positions with C*sp*^3^—H = 0.97 and O—H = 0.82Å and constrained to ride on their parent atoms, with *U*_iso_(H)=1.2*U*_eq_(1.5*U*_eq_for methyl H). H atoms attached to O(water) atoms of (I) were located from difference Fourier maps and refined with a global *U*_iso_ value.

### Crystal data

**Table:**

[KV(C_10_H_13_N_2_O_8_)O(H_2_O_1_)_3_]	*F*_000_ = 924
*M**_r_* = 449.31	*D*_x_ = 1.768 Mg m^−^^3^
Monoclinic, *P*2_1_/*c*	Mo *K*α radiation λ = 0.71073 Å
Hall symbol: -P 2ybc	Cell parameters from 3074 reflections
*a* = 6.6701 (13) Å	θ = 2.1–26.6º
*b* = 13.618 (3) Å	µ = 0.90 mm^−^^1^
*c* = 18.693 (4) Å	*T* = 298 (2) K
β = 96.150 (2)º	Block, blue
*V* = 1688.2 (6) Å^3^	0.40 × 0.30 × 0.20 mm
*Z* = 4	

### Data collection

**Table:**

Bruker SMART 1K CCD diffractometer	2957 independent reflections
Radiation source: fine-focus sealed tube	2613 reflections with *I* > 2σ(*I*)
Monochromator: graphite	*R*_int_ = 0.024
*T* = 298(2) K	θ_max_ = 25.0º
ω scans	θ_min_ = 2.7º
Absorption correction: multi-scan(SADABS; Sheldrick, 2000)	*h* = −7→7
*T*_min_ = 0.714, *T*_max_ = 0.840	*k* = −16→16
6813 measured reflections	*l* = −12→22

### Refinement

**Table:**

Refinement on *F*^2^	Secondary atom site location: difference Fourier map
Least-squares matrix: full	Hydrogen site location: inferred from neighbouring sites
*R*[*F*^2^ > 2σ(*F*^2^)] = 0.036	H-atom parameters constrained
*wR*(*F*^2^) = 0.088	*w* = 1/[σ^2^(*F*_o_^2^) + (0.0425*P*)^2^ + 0.6912*P*] where *P* = (*F*_o_^2^ + 2*F*_c_^2^)/3
*S* = 1.07	(Δ/σ)_max_ = 0.001
2957 reflections	Δρ_max_ = 0.33 e Å^−^^3^
236 parameters	Δρ_min_ = −0.22 e Å^−^^3^
Primary atom site location: structure-invariant direct methods	Extinction correction: none

### Special details

**Table:**

Geometry. All e.s.d.'s (except the e.s.d. in the dihedral angle between two l.s. planes) are estimated using the full covariance matrix. The cell e.s.d.'s are taken into account individually in the estimation of e.s.d.'s in distances, angles and torsion angles; correlations between e.s.d.'s in cell parameters are only used when they are defined by crystal symmetry. An approximate (isotropic) treatment of cell e.s.d.'s is used for estimating e.s.d.'s involving l.s. planes.
Refinement. Refinement of *F*^2^ against ALL reflections. The weighted *R*-factor *wR* and goodness of fit *S* are based on *F*^2^, conventional *R*-factors *R* are based on *F*, with *F* set to zero for negative *F*^2^. The threshold expression of *F*^2^ > σ(*F*^2^) is used only for calculating *R*-factors(gt) *etc*. and is not relevant to the choice of reflections for refinement. *R*-factors based on *F*^2^ are statistically about twice as large as those based on *F*, and *R*- factors based on ALL data will be even larger.

### Fractional atomic coordinates and isotropic or equivalent isotropic displacement parameters (Å^2^)

**Table:**

	*x*	*y*	*z*	*U*_iso_*/*U*_eq_	
V1	0.46422 (6)	1.03027 (3)	0.20517 (2)	0.02305 (14)	
N1	0.2470 (3)	0.90064 (14)	0.21295 (10)	0.0229 (4)	
N2	0.2025 (3)	1.08521 (14)	0.13790 (10)	0.0226 (4)	
C1	0.4610 (4)	0.85480 (18)	0.11714 (13)	0.0268 (5)	
C2	0.3056 (4)	0.82187 (17)	0.16472 (13)	0.0272 (5)	
H2A	0.1867	0.7994	0.1348	0.033*	
H2B	0.3589	0.7668	0.1936	0.033*	
C3	0.4930 (4)	0.88638 (17)	0.31905 (13)	0.0274 (6)	
C4	0.2772 (4)	0.86957 (19)	0.28900 (13)	0.0288 (6)	
H4A	0.2442	0.8005	0.2926	0.035*	
H4B	0.1883	0.9067	0.3167	0.035*	
C5	0.0432 (4)	0.93920 (17)	0.18987 (13)	0.0260 (5)	
H5A	−0.0044	0.9778	0.2283	0.031*	
H5B	−0.0499	0.8852	0.1789	0.031*	
C6	0.0520 (4)	1.00233 (18)	0.12396 (13)	0.0269 (5)	
H6A	−0.0806	1.0296	0.1095	0.032*	
H6B	0.0897	0.9620	0.0847	0.032*	
C7	0.1193 (4)	1.16469 (18)	0.18111 (13)	0.0281 (6)	
H7A	−0.0263	1.1660	0.1706	0.034*	
H7B	0.1711	1.2275	0.1669	0.034*	
C8	0.1722 (4)	1.15125 (17)	0.26067 (14)	0.0296 (6)	
C9	0.2569 (4)	1.12454 (18)	0.06851 (13)	0.0261 (5)	
H9A	0.3120	1.0715	0.0420	0.031*	
H9B	0.3622	1.1733	0.0785	0.031*	
C10	0.0834 (4)	1.17089 (18)	0.02109 (13)	0.0284 (6)	
K1	0.31886 (9)	0.85434 (4)	0.49345 (3)	0.03673 (17)	
O1	0.6281 (3)	1.11296 (13)	0.19480 (10)	0.0388 (5)	
O2	0.5346 (3)	0.94067 (12)	0.12620 (9)	0.0288 (4)	
O3	0.5115 (3)	0.79597 (13)	0.07183 (10)	0.0363 (4)	
O4	0.5988 (3)	0.94463 (12)	0.28376 (9)	0.0305 (4)	
O5	0.5578 (3)	0.84570 (13)	0.37597 (10)	0.0365 (4)	
O6	0.3143 (3)	1.08920 (12)	0.28047 (9)	0.0315 (4)	
O7	0.0813 (3)	1.19822 (14)	0.30238 (10)	0.0454 (5)	
O8	0.1269 (3)	1.20678 (14)	−0.04052 (9)	0.0370 (4)	
H8	0.2453	1.1952	−0.0455	0.056*	
O9	−0.0876 (3)	1.17595 (15)	0.03675 (10)	0.0412 (5)	
O10	0.6764 (3)	0.94372 (15)	0.56121 (11)	0.0502 (5)	
H10A	0.7731	0.9205	0.5434	0.075*	
H10B	0.6886	0.9215	0.6022	0.075*	
O11	0.1732 (3)	0.91295 (14)	0.61935 (10)	0.0474 (5)	
H11A	0.1017	0.8743	0.6387	0.071*	
H11B	0.2279	0.9442	0.6535	0.071*	
O12	−0.0659 (3)	0.90505 (16)	0.44337 (13)	0.0578 (6)	
H12A	−0.1676	0.8828	0.4205	0.087*	
H12B	−0.0626	0.9615	0.4282	0.087*	

### Atomic displacement parameters (Å^2^)

**Table:**

	*U*^11^	*U*^22^	*U*^33^	*U*^12^	*U*^13^	*U*^23^
V1	0.0205 (2)	0.0245 (2)	0.0242 (2)	−0.00226 (16)	0.00252 (16)	−0.00034 (16)
N1	0.0218 (11)	0.0252 (10)	0.0219 (10)	0.0000 (8)	0.0039 (8)	0.0015 (8)
N2	0.0232 (11)	0.0233 (10)	0.0221 (10)	0.0013 (8)	0.0054 (8)	0.0025 (8)
C1	0.0241 (13)	0.0295 (13)	0.0264 (13)	0.0031 (10)	0.0010 (10)	−0.0001 (11)
C2	0.0270 (13)	0.0224 (12)	0.0325 (14)	−0.0007 (10)	0.0042 (11)	−0.0018 (10)
C3	0.0305 (14)	0.0238 (12)	0.0278 (14)	0.0042 (11)	0.0029 (11)	0.0005 (11)
C4	0.0305 (14)	0.0316 (13)	0.0251 (13)	−0.0019 (11)	0.0062 (11)	0.0051 (11)
C5	0.0197 (12)	0.0272 (12)	0.0316 (14)	−0.0025 (10)	0.0044 (10)	−0.0008 (11)
C6	0.0218 (13)	0.0274 (12)	0.0306 (13)	−0.0036 (10)	−0.0017 (10)	−0.0005 (11)
C7	0.0272 (14)	0.0266 (13)	0.0314 (14)	0.0028 (10)	0.0072 (11)	−0.0028 (10)
C8	0.0315 (15)	0.0262 (13)	0.0328 (14)	−0.0052 (11)	0.0104 (11)	−0.0047 (11)
C9	0.0273 (13)	0.0277 (12)	0.0241 (12)	0.0005 (10)	0.0062 (10)	0.0016 (10)
C10	0.0296 (15)	0.0282 (13)	0.0269 (13)	−0.0011 (11)	0.0008 (11)	0.0002 (10)
K1	0.0338 (3)	0.0413 (3)	0.0348 (3)	0.0010 (3)	0.0025 (3)	0.0065 (3)
O1	0.0319 (11)	0.0358 (10)	0.0487 (12)	−0.0090 (8)	0.0045 (9)	−0.0002 (9)
O2	0.0296 (10)	0.0285 (9)	0.0300 (9)	−0.0030 (8)	0.0107 (8)	−0.0029 (7)
O3	0.0370 (11)	0.0372 (10)	0.0363 (10)	−0.0014 (8)	0.0108 (8)	−0.0116 (9)
O4	0.0261 (10)	0.0341 (9)	0.0306 (10)	−0.0011 (8)	0.0002 (7)	0.0061 (8)
O5	0.0387 (11)	0.0381 (10)	0.0311 (10)	0.0021 (8)	−0.0033 (8)	0.0072 (8)
O6	0.0385 (11)	0.0340 (9)	0.0226 (9)	0.0048 (8)	0.0055 (8)	−0.0027 (7)
O7	0.0574 (13)	0.0446 (11)	0.0372 (11)	0.0087 (10)	0.0187 (10)	−0.0100 (9)
O8	0.0336 (11)	0.0506 (12)	0.0272 (10)	0.0050 (9)	0.0050 (8)	0.0086 (9)
O9	0.0268 (11)	0.0532 (12)	0.0443 (12)	0.0067 (9)	0.0071 (9)	0.0162 (9)
O10	0.0508 (13)	0.0523 (12)	0.0461 (12)	−0.0007 (10)	−0.0017 (10)	0.0093 (10)
O11	0.0568 (14)	0.0454 (12)	0.0398 (12)	−0.0081 (10)	0.0037 (10)	0.0008 (9)
O12	0.0492 (14)	0.0465 (12)	0.0727 (16)	−0.0020 (10)	−0.0155 (11)	0.0145 (11)

### Geometric parameters (Å, °)

**Table:**

V1—O1	1.5955 (18)	C7—H7A	0.9700
V1—O6	1.9815 (17)	C7—H7B	0.9700
V1—O2	2.0092 (16)	C8—O7	1.219 (3)
V1—O4	2.0104 (17)	C8—O6	1.294 (3)
V1—N2	2.172 (2)	C9—C10	1.518 (3)
V1—N1	2.298 (2)	C9—H9A	0.9700
N1—C4	1.476 (3)	C9—H9B	0.9700
N1—C5	1.478 (3)	C10—O9	1.210 (3)
N1—C2	1.481 (3)	C10—O8	1.312 (3)
N2—C9	1.484 (3)	K1—O12	2.725 (2)
N2—C7	1.493 (3)	K1—O3^i^	2.7539 (19)
N2—C6	1.514 (3)	K1—O11	2.758 (2)
C1—O3	1.238 (3)	K1—O10	2.851 (2)
C1—O2	1.272 (3)	K1—O5	2.851 (2)
C1—C2	1.505 (3)	K1—O9^ii^	2.900 (2)
C2—H2A	0.9700	K1—O10^iii^	2.935 (2)
C2—H2B	0.9700	K1—K1^iii^	4.6380 (14)
C3—O5	1.236 (3)	K1—H12B	3.0701
C3—O4	1.289 (3)	O3—K1^iv^	2.7539 (19)
C3—C4	1.505 (4)	O8—H8	0.8200
C4—H4A	0.9700	O9—K1^v^	2.900 (2)
C4—H4B	0.9700	O10—K1^iii^	2.935 (2)
C5—C6	1.509 (3)	O10—H10A	0.8199
C5—H5A	0.9700	O10—H10B	0.8200
C5—H5B	0.9700	O11—H11A	0.8200
C6—H6A	0.9700	O11—H11B	0.8200
C6—H6B	0.9700	O12—H12A	0.8200
C7—C8	1.502 (4)	O12—H12B	0.8200
			
O1—V1—O6	101.82 (9)	O6—C8—C7	116.7 (2)
O1—V1—O2	97.02 (8)	N2—C9—C10	114.7 (2)
O6—V1—O2	160.61 (7)	N2—C9—H9A	108.6
O1—V1—O4	103.90 (9)	C10—C9—H9A	108.6
O6—V1—O4	86.31 (7)	N2—C9—H9B	108.6
O2—V1—O4	93.63 (7)	C10—C9—H9B	108.6
O1—V1—N2	101.83 (9)	H9A—C9—H9B	107.6
O6—V1—N2	80.59 (7)	O9—C10—O8	119.6 (2)
O2—V1—N2	91.17 (7)	O9—C10—C9	124.2 (2)
O4—V1—N2	153.01 (7)	O8—C10—C9	116.2 (2)
O1—V1—N1	174.01 (9)	O12—K1—O3^i^	137.71 (6)
O6—V1—N1	84.06 (7)	O12—K1—O11	79.34 (7)
O2—V1—N1	77.21 (7)	O3^i^—K1—O11	87.02 (6)
O4—V1—N1	75.13 (7)	O12—K1—O10	139.18 (7)
N2—V1—N1	80.10 (7)	O3^i^—K1—O10	76.15 (6)
C4—N1—C5	114.18 (18)	O11—K1—O10	81.47 (7)
C4—N1—C2	111.16 (19)	O12—K1—O5	109.24 (7)
C5—N1—C2	111.99 (19)	O3^i^—K1—O5	96.41 (5)
C4—N1—V1	105.01 (14)	O11—K1—O5	161.12 (6)
C5—N1—V1	105.97 (13)	O10—K1—O5	81.36 (6)
C2—N1—V1	107.95 (14)	O12—K1—O9^ii^	71.57 (6)
C9—N2—C7	110.56 (18)	O3^i^—K1—O9^ii^	71.89 (6)
C9—N2—C6	109.77 (18)	O11—K1—O9^ii^	100.37 (6)
C7—N2—C6	110.82 (18)	O10—K1—O9^ii^	147.80 (6)
C9—N2—V1	111.95 (14)	O5—K1—O9^ii^	98.34 (5)
C7—N2—V1	105.00 (14)	O12—K1—O10^iii^	71.81 (6)
C6—N2—V1	108.65 (14)	O3^i^—K1—O10^iii^	149.29 (6)
O3—C1—O2	123.8 (2)	O11—K1—O10^iii^	92.51 (6)
O3—C1—C2	117.8 (2)	O10—K1—O10^iii^	73.43 (7)
O2—C1—C2	118.4 (2)	O5—K1—O10^iii^	75.09 (6)
N1—C2—C1	112.82 (19)	O9^ii^—K1—O10^iii^	137.93 (6)
N1—C2—H2A	109.0	O12—K1—K1^iii^	105.52 (5)
C1—C2—H2A	109.0	O3^i^—K1—K1^iii^	113.39 (5)
N1—C2—H2B	109.0	O11—K1—K1^iii^	86.36 (5)
C1—C2—H2B	109.0	O10—K1—K1^iii^	37.34 (4)
H2A—C2—H2B	107.8	O5—K1—K1^iii^	75.22 (4)
O5—C3—O4	123.8 (2)	O9^ii^—K1—K1^iii^	171.84 (5)
O5—C3—C4	119.0 (2)	O10^iii^—K1—K1^iii^	36.09 (4)
O4—C3—C4	117.2 (2)	O12—K1—H12B	14.8
N1—C4—C3	110.02 (19)	O3^i^—K1—H12B	152.2
N1—C4—H4A	109.7	O11—K1—H12B	81.3
C3—C4—H4A	109.7	O10—K1—H12B	126.3
N1—C4—H4B	109.7	O5—K1—H12B	102.8
C3—C4—H4B	109.7	O9^ii^—K1—H12B	85.4
H4A—C4—H4B	108.2	O10^iii^—K1—H12B	57.0
N1—C5—C6	109.02 (19)	K1^iii^—K1—H12B	91.1
N1—C5—H5A	109.9	C1—O2—V1	122.60 (15)
C6—C5—H5A	109.9	C1—O3—K1^iv^	134.36 (16)
N1—C5—H5B	109.9	C3—O4—V1	120.32 (16)
C6—C5—H5B	109.9	C3—O5—K1	118.15 (16)
H5A—C5—H5B	108.3	C8—O6—V1	118.11 (15)
C5—C6—N2	111.54 (19)	C10—O8—H8	109.5
C5—C6—H6A	109.3	C10—O9—K1^v^	119.74 (16)
N2—C6—H6A	109.3	K1—O10—K1^iii^	106.57 (7)
C5—C6—H6B	109.3	K1—O10—H10A	108.2
N2—C6—H6B	109.3	K1^iii^—O10—H10A	100.5
H6A—C6—H6B	108.0	K1—O10—H10B	104.6
N2—C7—C8	112.7 (2)	K1^iii^—O10—H10B	131.8
N2—C7—H7A	109.1	H10A—O10—H10B	103.5
C8—C7—H7A	109.1	K1—O11—H11A	117.7
N2—C7—H7B	109.1	K1—O11—H11B	130.3
C8—C7—H7B	109.1	H11A—O11—H11B	102.8
H7A—C7—H7B	107.8	K1—O12—H12A	141.4
O7—C8—O6	124.0 (2)	K1—O12—H12B	107.3
O7—C8—C7	119.3 (2)	H12A—O12—H12B	102.5
			
O6—V1—N1—C4	−57.66 (15)	N2—C7—C8—O7	−165.0 (2)
O2—V1—N1—C4	127.40 (15)	N2—C7—C8—O6	15.1 (3)
O4—V1—N1—C4	30.08 (14)	C7—N2—C9—C10	−59.1 (3)
N2—V1—N1—C4	−139.11 (15)	C6—N2—C9—C10	63.5 (2)
O6—V1—N1—C5	63.53 (14)	V1—N2—C9—C10	−175.79 (16)
O2—V1—N1—C5	−111.41 (15)	N2—C9—C10—O9	−0.5 (4)
O4—V1—N1—C5	151.27 (15)	N2—C9—C10—O8	179.7 (2)
N2—V1—N1—C5	−17.92 (14)	O3—C1—O2—V1	−175.28 (19)
O6—V1—N1—C2	−176.33 (15)	C2—C1—O2—V1	3.9 (3)
O2—V1—N1—C2	8.74 (14)	O1—V1—O2—C1	171.07 (19)
O4—V1—N1—C2	−88.59 (15)	O6—V1—O2—C1	−22.6 (3)
N2—V1—N1—C2	102.23 (15)	O4—V1—O2—C1	66.57 (19)
O1—V1—N2—C9	42.20 (16)	N2—V1—O2—C1	−86.86 (19)
O6—V1—N2—C9	142.42 (16)	N1—V1—O2—C1	−7.28 (18)
O2—V1—N2—C9	−55.22 (15)	O2—C1—O3—K1^iv^	−150.33 (18)
O4—V1—N2—C9	−155.57 (16)	C2—C1—O3—K1^iv^	30.5 (3)
N1—V1—N2—C9	−132.03 (15)	O5—C3—O4—V1	−165.98 (18)
O1—V1—N2—C7	−77.80 (15)	C4—C3—O4—V1	12.8 (3)
O6—V1—N2—C7	22.42 (14)	O1—V1—O4—C3	161.53 (18)
O2—V1—N2—C7	−175.22 (14)	O6—V1—O4—C3	60.26 (18)
O4—V1—N2—C7	84.4 (2)	O2—V1—O4—C3	−100.31 (18)
N1—V1—N2—C7	107.97 (14)	N2—V1—O4—C3	−0.5 (3)
O1—V1—N2—C6	163.60 (15)	N1—V1—O4—C3	−24.57 (17)
O6—V1—N2—C6	−96.18 (14)	O4—C3—O5—K1	131.8 (2)
O2—V1—N2—C6	66.17 (14)	C4—C3—O5—K1	−46.9 (3)
O4—V1—N2—C6	−34.2 (2)	O12—K1—O5—C3	17.34 (19)
N1—V1—N2—C6	−10.63 (14)	O3^i^—K1—O5—C3	163.20 (17)
C4—N1—C2—C1	−124.2 (2)	O11—K1—O5—C3	−97.2 (2)
C5—N1—C2—C1	106.8 (2)	O10—K1—O5—C3	−121.92 (18)
V1—N1—C2—C1	−9.5 (2)	O9^ii^—K1—O5—C3	90.64 (18)
O3—C1—C2—N1	−175.9 (2)	O10^iii^—K1—O5—C3	−46.87 (17)
O2—C1—C2—N1	4.9 (3)	K1^iii^—K1—O5—C3	−84.25 (17)
C5—N1—C4—C3	−148.1 (2)	O7—C8—O6—V1	−173.6 (2)
C2—N1—C4—C3	84.1 (2)	C7—C8—O6—V1	6.3 (3)
V1—N1—C4—C3	−32.4 (2)	O1—V1—O6—C8	83.24 (19)
O5—C3—C4—N1	−164.2 (2)	O2—V1—O6—C8	−82.9 (3)
O4—C3—C4—N1	17.0 (3)	O4—V1—O6—C8	−173.32 (18)
C4—N1—C5—C6	158.6 (2)	N2—V1—O6—C8	−17.00 (17)
C2—N1—C5—C6	−73.9 (2)	N1—V1—O6—C8	−97.91 (18)
V1—N1—C5—C6	43.5 (2)	O8—C10—O9—K1^v^	−41.5 (3)
N1—C5—C6—N2	−57.1 (3)	C9—C10—O9—K1^v^	138.70 (19)
C9—N2—C6—C5	161.47 (19)	O12—K1—O10—K1^iii^	−32.58 (13)
C7—N2—C6—C5	−76.1 (2)	O3^i^—K1—O10—K1^iii^	175.77 (8)
V1—N2—C6—C5	38.7 (2)	O11—K1—O10—K1^iii^	−95.21 (7)
C9—N2—C7—C8	−146.5 (2)	O5—K1—O10—K1^iii^	76.92 (7)
C6—N2—C7—C8	91.6 (2)	O9^ii^—K1—O10—K1^iii^	168.86 (8)
V1—N2—C7—C8	−25.6 (2)	O10^iii^—K1—O10—K1^iii^	0.0

Symmetry codes: (i) *x*, −*y*+3/2, *z*+1/2; (ii) −*x*, *y*−1/2, −*z*+1/2; (iii) −*x*+1, −*y*+2, −*z*+1; (iv) *x*, −*y*+3/2, *z*−1/2; (v) −*x*, *y*+1/2, −*z*+1/2.

### Hydrogen-bond geometry (Å, °)

**Table:**

*D*—H···*A*	*D*—H	H···*A*	*D*···*A*	*D*—H···*A*
O8—H8···O3^vi^	0.82	1.75	2.542 (3)	162
O12—H12B···O11^vii^	0.82	2.03	2.802 (3)	157
O11—H11B···O4^iii^	0.82	2.17	2.960 (3)	162
O10—H10B···O6^iii^	0.82	2.20	2.987 (3)	161
O12—H12A···O5^viii^	0.82	1.99	2.804 (3)	169
O11—H11A···O7^vii^	0.82	1.99	2.801 (3)	169
O10—H10A···O12^ix^	0.82	2.26	2.983 (3)	147

Symmetry codes: (vi) −*x*+1, −*y*+2, −*z*; (vii) −*x*, −*y*+2, −*z*+1; (iii) −*x*+1, −*y*+2, −*z*+1; (viii) *x*−1, *y*, *z*; (ix) *x*+1, *y*, *z*.
